# Financial text analysis and credit risk assessment using a GPT-4 and improved BERT fusion model

**DOI:** 10.1371/journal.pone.0336217

**Published:** 2025-11-18

**Authors:** Huirong Tan, Yanruixue Xie

**Affiliations:** 1 School of Economics and Management, Shanghai Zhongqiao Vocational and Technical University, Shanghai City, China; 2 School of Business Administration, Southwestern University of Finance and Economics, Chengdu City, Sichuan, China; University of Sargodha, PAKISTAN

## Abstract

This study aims to improve the identification of potential credit risks in unstructured financial texts. It addresses the core problem of financial text analysis and credit risk assessment by proposing a hybrid model that combines the generative semantic understanding of Generative Pre-trained Transformer-4 (GPT-4) with the enhanced feature extraction of Bidirectional Encoder Representations from Transformers (BERT). To overcome the limitations of traditional methods—such as weak contextual reasoning in long texts, insufficient recognition of industry-specific terminology, and implicit credit risk expressions—the model incorporates a financial dictionary enhancement module and a named entity recognition (NER) component. GPT-4 is leveraged for prompt-based generation to extract latent risk information from complex texts, including annual reports. A dual-model semantic fusion mechanism with attention weighting constructs a multi-level risk assessment system that integrates contextual understanding, industry adaptability, and interpretability. Experiments on multiple publicly available financial datasets and real-world annual reports demonstrate the model’s effectiveness. Results show that the proposed approach outperforms representative baseline models in accuracy, adaptability, and interpretability. This work carries both theoretical and practical significance for research at the intersection of financial technology and natural language processing.

## Introduction

The rapid advancement of artificial intelligence, especially in natural language processing (NLP), is transforming data analysis in the financial sector. Large volumes of unstructured textual data—such as company annual reports, financial announcements, news articles, and investment commentaries—contain critical information about corporate performance and credit risk [1,2]. Efficiently extracting value from these texts to support risk identification and informed decision-making has become a major focus in fintech research [3]. Traditional credit risk assessment methods mainly rely on financial indicators, structured data models, or expert judgment. While these methods can be useful in specific scenarios, they often fail to capture the semantic features, sentiment tendencies, and latent risk signals embedded in text [4,5]. Pre-trained language models (PLMs) such as Bidirectional Encoder Representations from Transformers (BERT) and Generative Pre-trained Transformer (GPT) offer new solutions by leveraging contextual modeling and knowledge transfer. GPT-4, released in 2023, demonstrates exceptional capabilities in text generation, knowledge reasoning, and task understanding within complex contexts [6]. Despite these advances, significant challenges remain. Single-model approaches often struggle with highly specialized financial texts, limiting their ability to accurately recognize industry terminology and implicit risks. Their contextual reasoning in long-text scenarios is also constrained, which can result in overlooked risk information. Moreover, existing methods often lack sufficient interpretability and adaptability to specific financial contexts [7]. These issues create challenges for financial institutions seeking to directly apply pre-trained models to risk management.

To address these limitations, this study develops a hybrid model that combines GPT-4 with an enhanced BERT architecture for financial text analysis and credit risk assessment. By integrating GPT-4’s generative semantic modeling with BERT’s discriminative feature extraction, the model improves long-text reasoning, specialized vocabulary recognition, and risk classification. This enables precise identification of latent risk factors and supports scientifically grounded credit rating assessments.

The main contributions of this study are:

(1) A framework that leverages both generative and discriminative model strengths, addressing the limitations of single-model approaches.
(2) Integration of a financial dictionary enhancement module and Named Entity Recognition (NER), improving adaptability to specialized financial contexts.
(3) A multi-level semantic fusion mechanism with attention weighting, enhancing both accuracy and interpretability of risk identification.
(4) Validation of the approach on multiple public financial datasets and real-world annual report scenarios, demonstrating practical potential for financial risk management applications.

### Literature review

With the development of artificial intelligence and big data technologies, research on financial text processing and credit risk assessment has expanded in both academia and industry. This progress has been particularly supported by advancements in NLP and deep learning models. This field has been continuously expanding. Fei et al. (2023) proposed that financial texts had a unique advantage in revealing a company’s credit status. Through techniques such as sentiment analysis and topic extraction, financial texts can effectively support credit rating systems [8]. Mengelkamp et al. (2022) constructed a risk dictionary based on multi-source financial texts, combining textual data with financial indicators for credit evaluation. Their experiments showed that text information significantly improved the accuracy of risk warning systems [9]. Xu et al. (2025) conducted a quasi-natural experiment based on China’s Green Finance Reform and Innovation Pilot Zones to examine the causal effect of green finance policies on inclusive green growth. They found that these policies significantly promoted inclusive green growth, and the conclusions remained robust across multiple sensitivity tests, highlighting the critical role of institutional and policy environments in financial development and risk governance [10]. Jin et al. (2024) proposed an information-fusion-based conceptual design decision method for patent texts, integrating “incomplete evaluative semantics” and “plan belief.” Empirical results demonstrated that this approach enhanced decision robustness and interpretability under conditions of semantic uncertainty and incompleteness, providing a transferable technical pathway for knowledge extraction and fusion from complex texts [11]. Lu and Wu (2025) systematically reviewed “intelligent auditing” from a data science perspective, showing that machine learning, NLP, and big data analytics could improve risk identification and anomaly detection. They also outlined paradigm shifts and framework evolution, offering methodological guidance for implementing intelligent systems in compliance auditing and financial risk management [12]. Yang et al. (2023) introduced an evidence-fusion method in multi-source information integration based on Gaussian kernel similarity that accounted for focal element belief weights. They demonstrated that this similarity measure exhibited boundedness, consistency, and symmetry when handling conflicting and incomplete information, effectively enhancing the stability and reliability of fusion results [13].

In summary, existing research has made significant progress in financial text analysis, the application of pre-trained language models, and multi-model fusion. However, several limitations remain. Most studies rely on a single language model, which cannot simultaneously address the needs of text generation and feature extraction. In addition, mainstream language models often struggle to adapt to financial terminology and context, reducing their accuracy in real-world applications. To address these issues, this study proposes a fusion model that combines GPT-4 with an improved BERT. The model integrates both language understanding and generation capabilities to enhance comprehensive perception of financial semantics. Furthermore, mechanisms such as financial dictionaries and industry entity recognition are incorporated, improving the model’s adaptation to the specific characteristics of financial domain texts.

### Research design

#### Model design approach.

In credit risk assessment based on financial texts, the accuracy of semantic understanding and the effective use of contextual information are key to model performance. To address the challenges of specialized terminology, complex structures, and implicit meanings in financial texts, this study develops a multi-level analysis framework that integrates GPT-4 with an enhanced BERT model. By combining the strengths of both pre-trained language models, this design aims to improve the model’s ability to identify credit risk information. The overall design approach is shown in Table 1:

**Table 1:** Model Design Analysis.

Design Approach	Analysis
Incorporating BERT as the Feature Extraction Base Model	BERT’s bidirectional encoding enables detailed contextual representation, making it well-suited for tasks such as classification and NER [14]. To address the specialization of financial texts and the diversity of tasks, this study fine-tunes the original BERT model and incorporates a financial dictionary embedding module. This enhancement improves the model’s ability to recognize industry-specific terminology and identify risk-related keywords.
Incorporating GPT-4 for Contextual Enhancement and Generative Semantic Understanding	As the latest generative language model, GPT-4 demonstrates significant advantages in capturing long-text semantics, reasoning logical relationships, and language generation [15]. This study utilizes GPT-4 to perform extensible semantic modeling of financial texts, such as refining risk descriptions, summarizing credit trends, and predicting potential credit issues, thereby providing multi-dimensional auxiliary information for risk assessment.
Fusion of BERT and GPT-4 Outputs to Construct a Multi-Level Decision Mechanism	In the model fusion strategy, this study adopts a “feature-level fusion + decision-level fusion” approach: First, the textual vectors output by the improved BERT model are concatenated with the semantic feature vectors generated by GPT-4 to form a unified risk feature space [16,17]. Then, a Fusion Attention Mechanism is introduced to dynamically adjust the weight distribution of outputs from different models. Finally, the risk levels are accurately outputted in the classification layer. This fusion model combines generation and discrimination, as well as perception and reasoning. It is based on a thorough exploration of textual semantic information. The model adapts to the diversity, specialization, and complexity of financial texts. As a result, it provides more intelligent and accurate support for credit risk assessment.

This fusion model design, based on a thorough exploration of textual semantic information, achieves an organic integration of generation and discrimination, perception and reasoning. It is tailored to meet the diverse, specialized, and complex nature of financial texts, providing more intelligent and accurate model support for credit risk assessment.

### Optimization of the BERT and GPT-4 fusion design

Although the original BERT model demonstrates strong capabilities in semantic representation, it exhibits certain limitations when applied to the financial domain, such as insufficient understanding of industry-specific terminology and imprecise entity recognition [18,19]. To address these shortcomings, this study implements several structural and semantic modeling improvements.

A domain-specific corpus comprising financial annual reports, announcements, and industry research reports is constructed to perform further pre-training of the model. This enables the model to better capture the unique linguistic structures and semantic expressions characteristic of financial texts. Additionally, a financial dictionary is incorporated to enhance the model’s sensitivity to high-frequency risk terms, financial terminology, and descriptions of credit events. Through dictionary-based embedding, the model focuses more effectively on keywords closely related to risk identification [20,21].

Following BERT’s output layer, a NER module is added to automatically extract critical information, such as company names, financial indicators, and credit events. This module provides structured input for downstream risk classification tasks, improving accuracy in information extraction and feature representation.

During the model fusion stage, an attention-guided feature aggregation mechanism is designed. Unlike traditional feature concatenation methods, this mechanism dynamically optimizes the weighting of BERT and GPT-4 output vectors, enabling more precise semantic integration. The deep semantic representations from the enhanced BERT are first combined with the contextual summaries generated by GPT-4. This concatenated vector is then mapped through a fully connected layer to produce a unified risk assessment vector [22]. An attention scoring mechanism then weights the feature vectors from the two sub-models, amplifying the expression of key features in the final classification.

The fused vector is finally input into a hierarchical classification structure. The first layer determines whether the text exhibits risk tendencies, while the second layer further categorizes the level of risk. This design allows the model to maintain high overall identification accuracy while providing fine-grained differentiation across risk levels, thereby enhancing both the practicality and interpretability of credit risk assessment.

Through this optimization design, the fusion model developed in this study achieves stronger generalization in financial text semantic understanding and provides more accurate classification of potential credit risks. This provides more practical and operable technical support for real-world financial risk control applications.

### Credit risk assessment system construction

After completing the model design and optimization, this study constructs a credit risk assessment system tailored for real-world financial text analysis. The system centers on the fusion model of GPT-4 and the improved BERT, integrating multiple modules for data processing, semantic analysis, risk identification, and result output. Together, these modules form a comprehensive risk recognition workflow. The system comprises five core modules: Data Preprocessing, Semantic Modeling, Feature Fusion, Risk Assessment, and Result Display. Overall, the fusion model system covers the full process from text input to risk output. It balances theoretical modeling with practical deployment and provides technical support for building an efficient, intelligent, and interpretable credit risk assessment platform for the financial industry.

The Data Preprocessing Module collects, cleans, and formats raw financial texts. The workflow includes removing HyperText Markup Language (HTML) tags and stopwords, sentence segmentation and tokenization, NER, and constructing a financial domain dictionary matching matrix. These steps ensure professional consistency for downstream model input. Considering the prevalence of non-standard expressions and long-text structures in Chinese financial texts, a hybrid approach combining rule-based segmentation with dependency syntax analysis is adopted to improve corpus quality and sentence boundary accuracy. The Semantic Modeling Module consists of the enhanced BERT and GPT-4 models [23,24]. In the enhanced BERT, a financial dictionary embedding layer is added on top of the original token, position, and segment embeddings. This dictionary embedding generates vectors by matching text tokens with professional financial terms. The dictionary vectors are concatenated with the original BERT embeddings and projected to a unified dimension as follows:


$$E_{final}=W[E_{bert};E_{dict}]+b$$ (1)

$E_{dict}$ is the dictionary vector, $E_{bert}$ is the original embedding, W is the trainable projection matrix, bis the bias term, and $E_{final}$ is the final output vector. This design allows the model to dynamically adjust the weight of financial terminology in semantic modeling. The NER module identifies key entities such as company names, financial indicators, and debt terms. These entities are included as additional token-level features for the BERT encoding layer, enhancing its contextual representation for financial texts [25]. For GPT-4, prompt engineering guides generative understanding, as detailed in the GPT-4 Prompts Supplementary. The GPT-4 outputs are then converted into fixed-size semantic vectors using a Sentence-BERT encoder with a hidden size of 1024. These vectors are fused with the enhanced BERT outputs in the subsequent module. The Feature Fusion Module applies an attention-weighted fusion mechanism to integrate the two types of semantic features adaptively:


$$\alpha =softmaxW[H_{bert};H_{gpt}]+b$$ (2)

$$H_{fusion}=\alpha_{1}H_{bert}+\alpha_{2}H_{gpt}$$ (3)

In these formulas, α, $\alpha_{1}$ and $\alpha_{2}$ are learnable weights; $H_{bert}$ and $H_{gpt}$ are the output vectors of BERT and GPT-4, respectively; $H_{fusion}$ is the final fused vector. A multi-layer perceptron (MLP) with Dropout (rate 0.3) is included to enhance generalization and prevent overfitting. Attention weight distributions across different text scenarios can be visualized as heatmaps, revealing the relative contributions of generative and discriminative models. In the Credit Risk Assessment Module, the fused feature vectors are input into a multi-layer classifier to predict company credit risk levels [26]. The classifier supports multiple output modes, including binary classification (risk/no risk), three-class classification (low/medium/high risk), and multi-level ratings (AAA–CCC), which can be flexibly configured according to business requirements. To improve confidence in the predictions, a calibration mechanism using temperature scaling is applied to post-process the output probabilities, enhancing the interpretability of the results.

Finally, the Result Display and Feedback Module presents the assessment results through a visualization interface. Displayed content includes company basic information and text sources, highlighted risk-related words or sentences, predicted credit risk levels and probabilities, a GPT-4 summary, and a risk logic graph generated from the feature fusion mechanism. This design improves result interpretability and enhances the system’s usability for practical business applications.

### Experimental design

The datasets selected for this study include the *Financial Phrase Bank*, *Sentiment Analysis for Financial News*, and *Credit Risk Dataset*.

- The *Financial Phrase Bank* consists of 4,840 sentences from English financial news, each labeled with sentiment categories (positive, negative, or neutral). The dataset is divided based on the consensus of 5–8 annotators, making it suitable for sentiment classification tasks in the financial domain. The dataset can be directly accessed and downloaded from the Hugging Face platform.
- The *Sentiment Analysis for Financial News* dataset contains sentiment annotations for financial news headlines, designed to analyze sentiment from the perspective of retail investors. The dataset can be used to train models to predict the potential impact of news headlines on the market. The dataset can be directly accessed and downloaded from Kaggle.
- The Credit Risk Dataset simulates credit bureau data, containing clients’ credit histories, with the goal of predicting potential credit defaulters. It includes various features used for credit risk modeling and assessment. The dataset is also available for direct download on Kaggle.

The Financial Phrase Bank and Sentiment Analysis for Financial News datasets are textual corpora commonly used in financial sentiment analysis and risk-related NLP tasks. In this study, these datasets are used to fine-tune and evaluate the model, assessing its performance in sentiment classification and risk identification. The Credit Risk dataset, in contrast, is originally tabular, containing numerical and categorical features typical of credit bureau records rather than natural language text. To make it compatible with language models such as BERT and GPT-4, a text-based transformation is applied. Each record is converted into a sentence-like textual description. Key features—for example, “a client has X years of credit history, the current loan amount is Y, repayment status is Z…”—are concatenated into natural language inputs using templates. This approach enables the models to process the data effectively. Without this transformation, directly feeding tabular features into BERT or GPT-4 would be inappropriate. Therefore, the Credit Risk dataset is strictly used in its text-transformed form.

To ensure reproducibility and reliability, all datasets follow a fixed splitting strategy. Financial Phrase Bank and Sentiment Analysis for Financial News are divided into training, validation, and test sets at a 70%: 15%: 15% ratio. Stratified sampling ensures that class distributions remain consistent across subsets. The text-transformed Credit Risk dataset is split in the same 70%: 15%: 15% ratio, using a fixed random seed to maintain consistency across experiments. For experiments involving company annual reports, samples are divided by year and company. This assigns data from different periods and industries to training, validation, and test sets, preventing information leakage and enhancing model generalization. All experiments are repeated under these same splits to ensure stable and comparable results.

To ensure the stability and reproducibility of the experiments, the hardware configuration was carefully planned as follows:

- Processor model: Intel Xeon Gold 6226R @ 2.90GHz
- Graphics processor model: NVIDIA A100 80GB PCIe
- Memory capacity: Samsung DDR4 ECC Registered, 256GB
- Storage device: Samsung PM1733 NVMe, 3.2TB
- Operating system version: Ubuntu 20.04 LTS (64-bit)

The comparison models selected for this study include Financial Bidirectional Encoder Representations from Transformers (FinBERT), Robustly Optimized BERT Pretraining Approach for Financial Text (RoBERTa-Fin), Sentiment Knowledge Enhanced Pre-training for Financial Text (SKEP-Fin), Decoding-enhanced BERT with Disentangled Attention for Financial Text (DeBERTa-Fin), and GPT with Synthetic Financial Risk Summarization Network (GPT-SynNet). FinBERT builds on the original BERT architecture and is further trained on large-scale financial corpora, enhancing its domain adaptation for semantic understanding, sentiment recognition, and risk modeling in financial texts. RoBERTa-Fin employs RoBERTa’s optimization strategies—such as larger training datasets, dynamic masking, and extended training duration—and undergoes secondary pretraining on financial corpora, improving robustness and semantic modeling in complex financial contexts. SKEP-Fin incorporates sentiment knowledge during pretraining, allowing it to capture semantic text representations and identify implicit sentiment tendencies in financial texts, making it more sensitive to early credit risk signals. DeBERTa-Fin uses a disentangled attention mechanism and relative position encoding to better model long texts. In financial scenarios, it effectively captures cross-sentence dependencies in reports and announcements, making it suitable for lengthy annual report analysis. GPT-SynNet combines GPT’s generative pretraining with a financial risk synthetic summary network, enabling automatic summarization of potential risk factors in long texts and demonstrating strong generalization in risk identification tasks. Together, these models span financial-specific fine-tuned architectures, structure-optimized Transformers, sentiment-enhanced models, and generative semantic models. They are used to benchmark the proposed model across classification accuracy, risk identification capability, and depth of text comprehension.

### Algorithm evaluation

#### Performance analysis.

**Fig 1:**
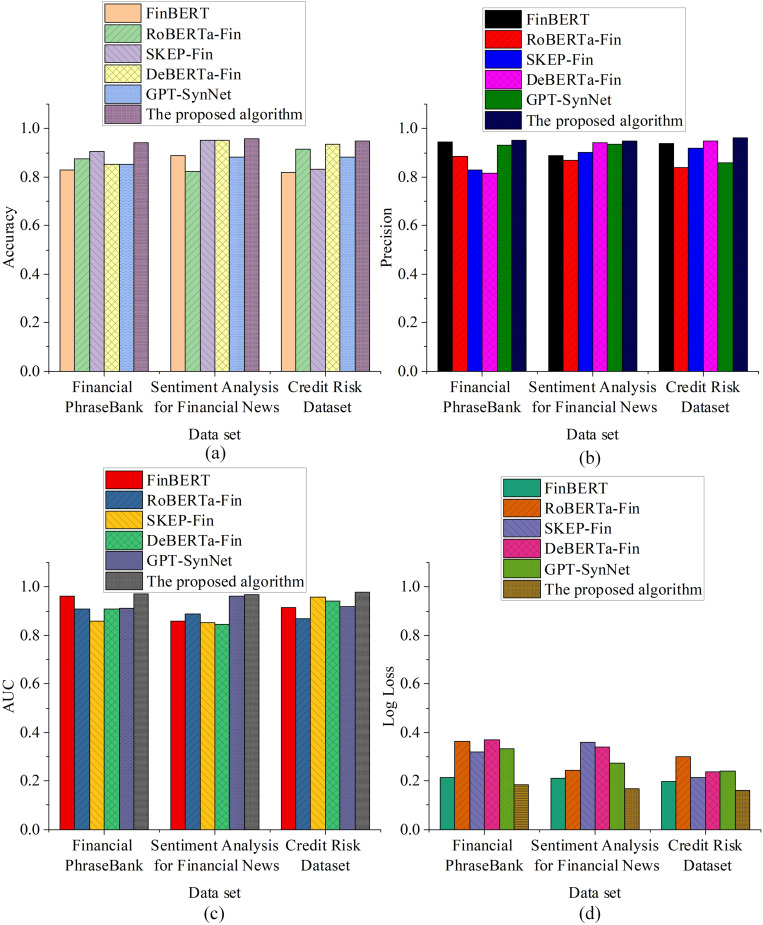
Comparative Evaluation of Classification Performance across Models(a) Accuracy results; (b) Precision results; (c) AUC comparison; (d) Log Loss assessment.

In the performance comparison experiment, this study evaluates the models from two perspectives: classification performance and generalization & stability. Each perspective includes four core metrics to ensure comprehensive and fair evaluation. The prediction performance metrics include accuracy, precision, Area Under the Curve (AUC), and logarithmic loss. The results of the classification performance evaluation are shown in Fig 1.

**Fig 2:**
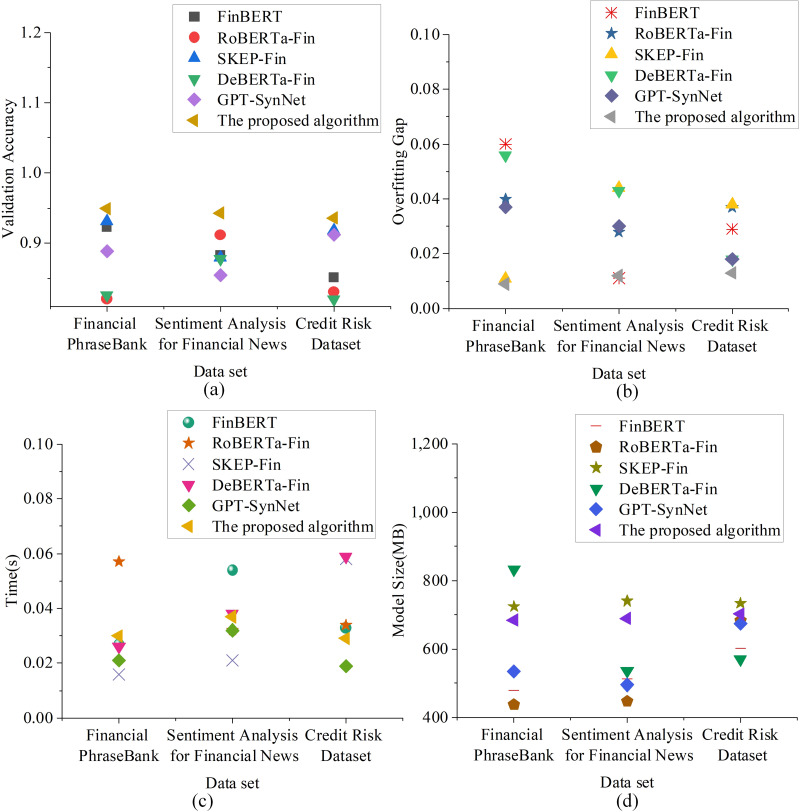
Assessment of Model Generalization Ability and Computational Stability(a) Validation accuracy trends; (b) Overfitting gap analysis; (c) Inference time per sample; (d) Model parameter size comparison.

The results in Fig 1 show that accuracy is the most fundamental classification performance metric. The proposed model achieves accuracy rates of 0.942, 0.957, and 0.948 on the three datasets, with none falling below 0.94, indicating stable and reliable performance across different text types. In comparison, FinBERT attains an accuracy of 0.821 on the Credit Risk dataset, while RoBERTa-Fin reaches 0.824 on the Sentiment Analysis dataset. These results underscore the superior adaptability of the proposed model to credit-related semantics. In terms of precision, the proposed model demonstrates a more sensitive ability to identify risks, with precision rates of 0.951, 0.947, and 0.962 across the three datasets. While GPT-SynNet performs reasonably well on the Sentiment Analysis dataset (0.934), it still lags slightly behind the proposed model. In contrast, RoBERTa-Fin’s precision on the Credit Risk dataset is 0.838, showing a significant performance gap. For AUC, the proposed model achieves 0.971, 0.967, and 0.978, with all scores approaching 1, indicating extremely high reliability in distinguishing between positive and negative samples. In comparison, DeBERTa-Fin achieves an AUC of only 0.847 on the Sentiment Analysis dataset, indicating a significant performance difference. Even though SKEP-Fin performs well on the Credit Risk dataset with an AUC of 0.958, it still falls short of the proposed model. In the Log Loss metric, the proposed model’s losses are 0.183, 0.167, and 0.159, the lowest among all models, indicating that its output probability distribution is more concentrated, with smaller errors. For example, SKEP-Fin has a Log Loss of 0.361 on the Sentiment Analysis dataset, nearly double that of the proposed model, showing greater uncertainty and predictive bias. The generalization and stability metrics include validation accuracy, overfitting gap, inference time per sample, and model parameter size. The evaluation results are shown in Fig 2:

The results in Fig 2 show that the proposed model’s validation accuracy is 0.949, 0.943, and 0.936, indicating that the model can maintain performance close to that during training without noticeable degradation. In contrast, DeBERTa-Fin achieves only 0.820 on the Credit Risk Dataset, suggesting weaker generalization capability. Regarding the overfitting gap, the proposed model exhibits values below 0.013 (0.009, 0.012, and 0.013 across the three datasets), substantially outperforming SKEP-Fin’s 0.044 on the Sentiment Analysis dataset. This indicates that the proposed model maintains consistent performance between training and validation, effectively avoiding common overfitting issues. In terms of inference efficiency, the proposed model’s inference time per sample is 0.030, 0.037, and 0.029 seconds. While slightly higher than some lightweight models (e.g., SKEP-Fin’s 0.016 seconds), it achieves a good balance between speed and performance while maintaining high precision. Lastly, in terms of model size, the proposed model is 685.357MB, 689.114MB, and 702.439MB in size, slightly larger than RoBERTa-Fin but smaller than DeBERTa-Fin (which reaches a maximum size of 832.360MB). However, it strikes a good balance between functional completeness, performance, and resource control, making it feasible for practical deployment.

To evaluate the independent contributions of key components to overall performance and stability, a systematic ablation study was conducted on the proposed fusion framework. The model variants are defined as follows:

A0: Full model. Enhanced BERT = base BERT + financial dictionary embedding + NER tags; GPT-4 branch generates summaries using fixed prompts → Sentence-BERT (1024-dim) vectorization; attention-weighted fusion; temperature scaling calibration.

A1: Removes financial dictionary embedding, all else unchanged.

A2: Removes NER tag features, all else unchanged.

A3: Removes GPT branch (only enhanced BERT remains), all else unchanged.

A4: Replaces attention fusion with simple concatenation + MLP (no α weights).

A5: Replaces attention fusion with equal-weight averaging.

A6: Disables temperature scaling (no confidence calibration) to observe its effect on calibration and threshold stability.

A7: Reduces GPT vector dimension from 1024 to 768, all else unchanged, to evaluate the impact of dimension on fusion gain and latency.

A8: Replaces enhanced BERT’s sentence vector from mean-pooling to [CLS] pooling.

Ablation results are shown in Table 2.

**Table 2:** Ablation Study Results.

Variant	PhraseBank Acc	PhraseBank F1	Financial News Acc	Financial News F1	Annual AUROC	ECE	Latency (s/doc)
A0	0.944 ± 0.006	0.943 ± 0.007	0.958 ± 0.005	0.956 ± 0.006	0.979 ± 0.004	0.021 ± 0.004	1.82
A1	0.934 ± 0.007	0.932 ± 0.008	0.948 ± 0.007	0.946 ± 0.007	0.969 ± 0.005	0.026 ± 0.005	1.78
A2	0.936 ± 0.006	0.935 ± 0.007	0.951 ± 0.006	0.949 ± 0.007	0.971 ± 0.005	0.025 ± 0.005	1.79
A3	0.929 ± 0.008	0.927 ± 0.009	0.944 ± 0.007	0.942 ± 0.008	0.955 ± 0.006	0.027 ± 0.006	1.12
A4	0.938 ± 0.006	0.937 ± 0.007	0.951 ± 0.006	0.950 ± 0.006	0.971 ± 0.005	0.024 ± 0.004	1.70
A5	0.936 ± 0.007	0.935 ± 0.007	0.949 ± 0.006	0.947 ± 0.006	0.968 ± 0.006	0.025 ± 0.005	1.69
A6	0.943 ± 0.006	0.942 ± 0.007	0.957 ± 0.005	0.955 ± 0.006	0.978 ± 0.004	0.067 ± 0.009	1.80
A7	0.941 ± 0.006	0.940 ± 0.007	0.955 ± 0.006	0.953 ± 0.006	0.974 ± 0.005	0.023 ± 0.004	1.62
A8	0.939 ± 0.007	0.938 ± 0.007	0.950 ± 0.006	0.949 ± 0.006	0.972 ± 0.005	0.024 ± 0.005	1.83

Table 2 shows that the full model achieves the highest or near-highest performance across all three datasets. On PhraseBank, accuracy and macro-F1 reach 0.944 and 0.943, respectively. On Financial News, accuracy and macro-F1 reach 0.958 and 0.956. On the annual report corpus, AUROC reaches 0.979, outperforming all other variants. Removing the financial dictionary embedding (A1) or NER features (A2) results in slight performance drops, with accuracy decreases of approximately 0.008–0.010 on PhraseBank and Financial News. Removing the GPT branch (A3) results in a more pronounced performance decline: AUROC on annual reports decreases to 0.955, while inference latency drops significantly, highlighting the generative branch’s substantial contribution to long-text risk modeling. Comparing fusion methods, attention-weighted fusion (A0) outperforms simple concatenation (A4) and equal-weight averaging (A5), both of which yield slightly lower performance than A0. Disabling temperature scaling (A6) has minimal effect on accuracy and F1 but significantly worsens the calibration metric ECE, which increases from 0.021 to 0.067. Reducing GPT vector dimensionality from 1024 to 768 (A7) causes a slight performance drop but improves inference latency. Using [CLS] pooling instead of mean-pooling (A8) also results in minor performance degradation. Robustness results are presented in Table 3.

**Table 3:** Robustness Evaluation Results.

Variant	Term Mask 10%	Term Mask 30%	Noise 3%	Annual Report Trim 2k	Annual Report Trim 5k
A0	1.8	5.6	2.1	6.9	3.2
A1	3.4	9.7	3.0	8.8	4.5
A2	3.1	9.1	2.9	8.2	4.2
A3	2.5	7.6	2.4	12.8	7.5
A4	2.6	7.8	2.6	9.5	5.0
A5	2.9	8.3	2.7	10.1	5.4
A6	1.9	5.8	2.2	7.1	3.3
A7	2.0	6.2	2.2	8.0	3.8
A8	2.3	7.1	2.5	8.6	4.1

Table 3 demonstrates that the full model (A0) exhibits the smallest performance drops under term masking, text noise, and annual report trimming scenarios (1.8%–6.9%), showing strong robustness. Removing the financial dictionary or NER features (A1/A2) leads to larger performance drops under term-masking conditions, particularly at 30% masking, with declines of 9.7% and 9.1%, respectively. Removing the GPT branch (A3) causes the most substantial decreases in long-text trimming scenarios, with 12.8% for 2k tokens and 7.5% for 5k tokens—far exceeding the full model’s drops. Replacing the fusion method (A4/A5) also reduces robustness across multiple perturbations, underscoring the advantage of attention-based fusion for maintaining stable performance.

### Company annual report credit analysis experiment

**Fig 3:**
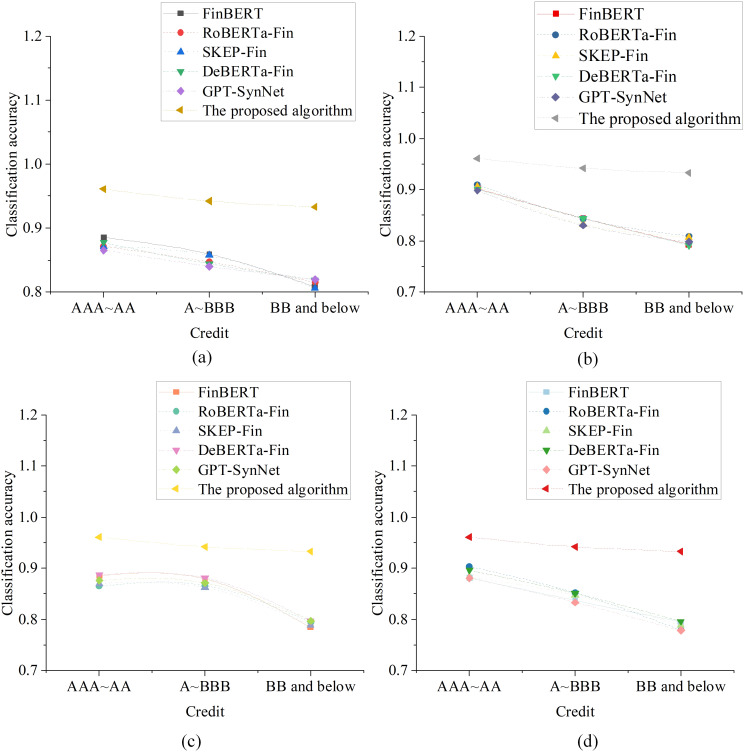
Case Study of Credit Risk Assessment Based on Annual Report Texts(a) Manufacturing sector company analysis; (b) Real estate sector company analysis; (c) Financial services sector company analysis; (d) Technology sector company analysis.

To further validate the effectiveness of the proposed fusion model in real-world financial text environments, a “Company Annual Report Credit Analysis Experiment” was designed using publicly disclosed annual reports of listed companies. This experiment aims to assess the model’s ability to understand semantics in complex, long text structures, identify credit risk factors, and accurately evaluate risk levels with interpretability. The experiment selected 800 annual reports from A-share companies listed on the main board from 2021 to 2023 as the text corpus, with risk labels mapped based on publicly available credit rating results. To ensure comprehensive data coverage and reasonable distribution, the selected companies span multiple industries, including manufacturing, real estate, financial services, and technology. The credit ratings are classified into three categories: high credit (A-AAAA), medium credit (A-BBB), and low credit (BB and below). The results of the experiment are shown in Fig 3.

The results in Fig 3 show that, overall, the optimized model achieves the highest classification accuracy across all three credit levels in every industry. In the manufacturing sector, the model recognizes high, medium, and low credit companies with accuracies of 0.961, 0.942, and 0.933, respectively. This demonstrates its strong adaptability to annual reports with clear structure and standardized language. In contrast, among the comparison models, DeBERTa-Fi only achieves a recognition accuracy of 0.819 for the low credit category, indicating certain blind spots in its identification ability. In the real estate industry, the optimized model maintains high accuracy with rates of 0.961, 0.942, and 0.933 for the three credit levels. This shows its robust performance in identifying reports from high-debt and high-volatile companies. However, GPT-SynNet shows a lower accuracy of 0.830 for medium credit companies, suggesting weaker stability in its recognition. In the financial services industry, where reports are dense with technical terms and complex structures, the proposed model continues to perform excellently, achieving accuracies of 0.961, 0.942, and 0.933 across the three credit levels. This highlights the model’s strong capabilities in multi-level semantic extraction and contextual integration. In comparison, FinBERT only reaches an accuracy of 0.784 for low credit identification, indicating a more conservative approach to risk detection. For technology companies’ annual reports, the optimized model again achieves accuracies of 0.961, 0.942, and 0.933 across the three credit levels. While RoBERTa-Fin performs better for high credit companies, its accuracy for the low credit category is only 0.779, with noticeable fluctuations in its overall performance.

## Discussion

The results from the performance comparison experiments clearly show that the proposed optimized model exhibits strong overall capabilities in understanding the semantics of financial texts, extracting risk features, and assessing credit levels. Unlike traditional BERT fine-tuning models, this model integrates the generative reasoning abilities of GPT-4 with the fine-grained feature encoding capabilities of the enhanced BERT. By doing so, it fully capitalizes on the strengths of language models in expressing various semantic levels. In terms of classification performance, the model demonstrates solid discriminative boundaries and stable risk identification. It consistently outperforms in key metrics such as accuracy and precision, suggesting its ability to make more informed judgments about the latent risk information embedded in financial texts. In the areas of generalization and stability, the model shows high robustness, with minimal performance differences between the training and validation sets. This indicates its suitability for different financial text contexts and its potential for cross-domain transfer. Additionally, its inference efficiency and model size are within acceptable limits, providing a solid foundation for practical deployment.

The results from the company annual report credit analysis experiment further highlight the performance of the proposed fusion model of GPT-4 and improved BERT. In the complex environment of financial annual reports, this model shows stable and highly distinguishable credit risk assessment capabilities. This advantage is evident not only in the improved classification metrics but also in the model’s adaptability to various company types, industry structures, and risk expressions. The model’s strong performance across different industries stems from its dual-model collaboration mechanism. The enhanced BERT module excels in extracting fine semantic details from financial terminology and structured descriptions, while GPT-4 enhances the model’s ability to perform contextual abstraction and logical reasoning in generative understanding. When these two models are combined, the model not only identifies explicit risk factors in texts but also perceives potential risks through language use and phrasing. Additionally, the model demonstrates high sensitivity in recognizing low-credit companies. It successfully detects potential risk signals such as “abnormal cash flow,” “decreased profitability,” and “management instability.” This capability is particularly valuable for financial institutions and rating agencies, as it aids in early-stage risk warnings. It is important to note that the performance of different models varies across industries. Traditional models are more susceptible to the structure and length of industry-specific texts, while the fusion model presented here exhibits strong robustness across diverse industries and structures. This stability indirectly validates the rationality and adaptability of the model’s design.

Integrating large-scale generative models, such as GPT-4, into the fusion framework indeed enhances semantic understanding and risk information aggregation in long texts. However, this integration inevitably introduces challenges in terms of computational cost and efficiency. From a computational perspective, using large-scale models requires more processing time and memory, resulting in significantly higher inference latency compared with solutions relying solely on discriminative models like BERT. Economically, reliance on commercial APIs can quickly accumulate token-based costs when processing large volumes of text, such as annual reports or financial disclosures, posing practical constraints on sustainable deployment. The impact of these issues varies across application scenarios. In real-time tasks, such as financial news monitoring or sentiment and risk alerts, inference latency can become a system bottleneck. Lightweight or hierarchical invocation strategies can help address this challenge. For instance, the BERT branch can be used for rapid initial screening, while the GPT branch is invoked only for low-confidence cases or highly complex texts. This approach reduces overall latency. In batch-processing scenarios, such as analyzing annual reports or industry reports, latency constraints are less strict. However, frequent GPT usage may still generate high costs, making it necessary to strike a careful balance between efficiency and expense.

## Conclusion

With the advancement of financial technology, large language models have become widely adopted in NLP. Efficiently analyzing financial texts and accurately identifying credit risks using deep semantic modeling techniques has now become a critical challenge in financial risk management. In response to this issue, this study proposes a financial text analysis and credit risk assessment model that combines the generative capabilities of GPT-4 with the encoding strengths of an improved BERT. The model is further optimized in terms of its structure, system design, and multi-dimensional empirical evaluation. The model architecture adopts a dual-model fusion framework that integrates BERT with GPT-4. An attention mechanism is employed for feature weighting and fusion, enhancing the understanding of financial terminology, latent risk-related language, and contextual dependencies. Methodological improvements include a financial dictionary embedding mechanism and an entity recognition module, which strengthen the domain adaptability of the enhanced BERT. Prompt engineering is further applied to improve the practicality and interpretability of GPT-4 in generating risk information summaries.

Although this study makes significant contributions, there are still some limitations due to time and resource constraints. Despite the substantial accuracy improvements, the model’s integration of two large pre-trained language models results in higher inference time and resource consumption. Future work will focus on exploring model compression and distillation techniques. The goal is to reduce computational resource demands and improve deployment efficiency, while ensuring the model’s effectiveness through knowledge distillation or lightweight structural designs.

## Supporting information

S1 File The data in the Figures can be found in the Supporting Information section ‘Data in Figures’. (ZIP)
